# Ionization inhibition in a polyol/water system for boosting H_2_ generation from NaBH_4_

**DOI:** 10.1039/d0ra07914f

**Published:** 2020-12-24

**Authors:** Lili Feng, Cong Wang, Jinrong Xu, Mingwei Fang, Yu Shi, Lei Xie, Jie Zheng, Xingguo Li

**Affiliations:** School of Chemical & Environmental Engineering, China University of Mining and Technology Beijing 100083 PR China; Beijing National Laboratory of Molecular Sciences, State Key Laboratory of Rare Earth Materials Chemistry and Applications, College of Chemistry and Molecular Engineering, Peking University Beijing 100871 PR China jiezheng@pku.edu.cn xgli@pku.edu.cn; Sunan Institute for Molecular Engineering, Peking University Changshu 215500 PR China

## Abstract

Alcoholysis and hydrolysis of NaBH_4_ to produce H_2_ offer attractive routes to sustainable development with high energy density and environmentally-friendly features. However, the productivity is often limited by the increased alkalinity of the reaction system and the deactivation of catalysts. Here, we present a novel strategy of constructing a polyol/water composite system to promote catalyst free alcoholysis of NaBH_4_ while inhibiting the ionization of reaction products. The polyol/water system exhibits a NaBH_4_ conversion of more than 90% in less than 60 min, especially when the erythritol/water system is employed with a conversion of 96% in 80 min. Further study shows that erythritol participates in the reaction and the ionization of reaction products is inhibited by erythritol. Moreover, the analysis of reaction products and control group results reveal that erythritol indeed inhibits the basicity enhancement of the reaction system *via* reacting with NaB(OH)_4_. By adjusting the volume of water in the polyol/water system, a quasi-solid phase reaction system is developed for practical applications, which shows an excellent NaBH_4_ conversion of 94% and high hydrogen storage gravimetric density of 3.9 wt%.

## Abstract 2

A different polyol/water system was constructed to promote the alcoholysis of NaBH_4_. The ionization of reaction products is inhibited, thus boosting H_2_ generation.

## Graphical abstract

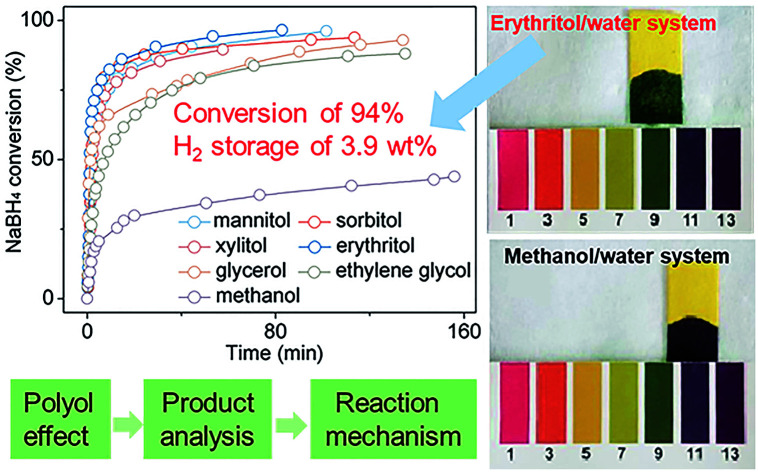

## 1. Introduction

Hydrogen (H_2_) is considered to be a promising energy carrier toward sustainable development with high energy density and environmentally-friendly features.^1^ Due to the gaseous nature and low density of H_2_ at room temperature, storage and transportation of H_2_ are still obstacles to the development of hydrogen energy.^2^ The controllable generation and purity of H_2_ have led to the hydrolysis of chemical hydrides being widely studied. Among them, sodium borohydride (NaBH_4_) deserves special attention owing to its high gravimetric hydrogen density (10.5 wt%), environmental friendliness and potential safe operation.^3,4^ The hydrolysis and alcoholysis of NaBH_4_ have been widely considered as promising methods for hydrogen generation.

Unfortunately, the hydrolysis of NaBH_4_ is always inhibited by the increased alkalinity of the solution resulting from the ionization of the reaction products. To solve this problem, a variety of catalysts have been developed for efficient hydrolysis of NaBH_4_, including metals, metal oxides, metal chalcogenides, carbon-based nanomaterials.^5–12^ The noble metals including Ru, Rh and Pt have been found to be the most efficient catalytic species, but their practical applications are restricted by the expensive price and limited resources.^13–15^ Moreover, earth abundant materials like Co, Ni, Fe show activities towards hydrolysis of NaBH_4_, while they suffer from slow kinetics and poor catalytic durability.^16–18^ For instance, Brown *et al.* have compared the catalytic activity of different metallic elements and observed that those metallic elements exert a catalytic effect on NaBH_4_ hydrolysis with an order: Ru, Rh > Pt > Co > Ni > Os > Ir > Fe > Pd.^19^ Besides, acids such as nitric acid and malic acid are also reported as catalysts to make the reaction more complete.^20^ The addition of acids can inhibit the ionization of reduction products, but causes environmental pollution.

Since the hydrolysis of NaBH_4_ is not efficient at low temperature, which may be inconvenient for automotive and portable applications.^21^ Alcoholysis of NaBH_4_ has been widely reported as another attractive route with fast reaction kinetics and low activation energy in comparison to hydrolysis of NaBH_4_. For example, Lo *et al.* reported that NaBH_4_ could generate H_2_ in methanol at low temperatures (253–323 K).^22^ Wang and co-workers adopted a strategy of constructing bimetallic catalytic system for boosting H_2_ generation rates from NaBH_4_ methanolysis. Significantly, the highest H_2_ generation rate is observed in the case of Ru–Co/C catalyst, achieving 9.36 L min^−1^ g^−1^ at 25 °C, which is comparable to the reported pure Ru catalysts.^23^ Although great progress has been achieved in improving the catalytic activity of catalysts for alcoholysis/hydrolysis of NaBH_4_, the increasing alkalinity of the solution resulting from the ionization of the reaction products is not fundamentally inhibited. Meanwhile, catalysts still confront great challenges in poor activity, low selectivity, and poor long-term stability. It is still far from satisfaction to achieve practical applications. Moreover, further studies are still necessary to elucidate its underlying mechanisms of alcoholysis/hydrolysis of NaBH_4_. Thus, rational design of reaction system is important to facilitate the alcoholysis/hydrolysis of NaBH_4_.

Here, we present a novel strategy of constructing a polyol/water composite system to promote the alcoholysis of NaBH_4_ without any catalyst while inhibiting the ionization of reaction products. The polyol/water system witnesses a NaBH_4_ conversion of more than 90% in less than 60 min, especially when the erythritol/water system is employed with a conversion of 96% in 80 min. Further study shows that erythritol participates in the reaction and the ionization of reaction products is inhibited by erythritol. Moreover, the analysis of reaction products and control group results further reveal that erythritol indeed inhibit the basicity enhancement of the reaction system *via* reacting with NaB(OH)_4_. By adjusting the volume of water in polyol/water system, a quasi-solid phase reaction system is developed for practical applications, which shows an excellent NaBH_4_ conversion of 94% and high hydrogen storage gravimetric density of 3.9 wt%.

## 2. Experimental

### 2.1 Materials

Sodium borohydride (NaBH_4_, 98%, Aldrich), methanol (CH_3_OH, 99%, Aldrich), ethylene glycol (C_2_H_6_O_2_, 99%, Aldrich), glycerol (C_3_H_8_O_3_, 99%, Aldrich), erythritol (C_4_H_10_O_4_, 99%, Adamas), xylitol (C_5_H_12_O_5_, 99%, Adamas), sorbitol (C_6_H_14_O_6_, 99%, Adamas), mannitol (C_6_H_14_O_6_, 99%, Adamas) and sodium metaborate (NaB(OH)_4_·2H_2_O, 99%, Adamas) were all analytical grade and used without further purification.

### 2.2 Hydrogen generation

The experiment was inducted in a flask containing NaBH_4_, polyols and water. Methanol, ethylene glycol, glycerol, erythritol, xylitol, sorbitol and mannitol were adopted as the polyol feedstock and tested individually. The reaction temperature was controlled by a oil bath with magnetic agitation. Upon H_2_ production from the reaction, the clean/pure H_2_ can replace the water that was filled in an inverted cylinder to determine the exact volume of the generated H_2_ from the reaction. To evaluate the reaction performances of different polyol/water composite systems, NaBH_4_ conversion was adopted as the evaluation index in this work, which was equal to H_2_ productivity and can be calculated according to the following equation:

1 NaBH_4_ conversion (%) = (*V*_r_/*V*_t_) × 100%

2 *V*_t_ = (*W*_NaBH_4__/*M*_NaBH_4__) × (4/1) × 24.5 (L mol^−1^ at 298 K)

where *V*_r_ (L) is the real volume of hydrogen, *V*_t_ (L) is the theoretical volume of hydrogen, and *W*_NaBH_4__ (g) and *M*_NaBH_4__ (g mol^−1^) are the weight and molecular weight of NaBH_4_, respectively.

### 2.3 Characterization

The chemical of reaction products were confirmed by the Fourier transform infrared (FTIR) spectra at 4 cm^−1^ resolution in the spectral range of 4000–500 cm^−1^ by FTIR spectrometer (Bruker, Tensor 27). The crystallization phase of reaction products was probed by X-ray powder diffraction (XRD) obtained using a Rigaku RX III powder diffractometer with a Cu Kα radiation. Moreover, the chemistry of reaction products was measured with a nuclear magnetic resonance (NMR) which was recorded in D_2_O at 500 MHz on a Bruker AV-400 NMR spectrometer.

## 3. Results and discussion

### 3.1 Hydrogen generation in different polyol/water system

**Fig. 1:**
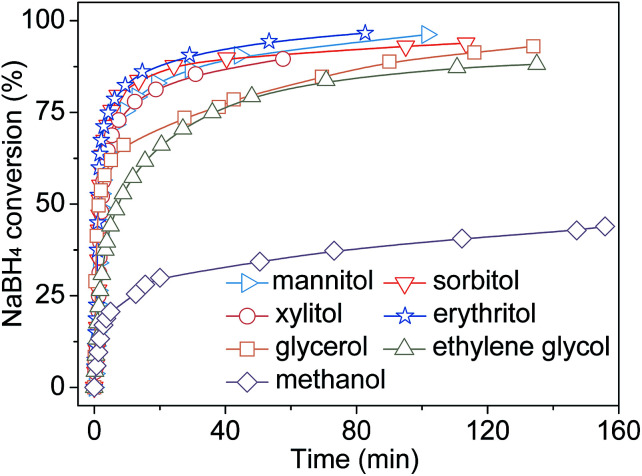
The conversion of NaBH_4_ in different polyol/water system. Mole ratio of NaBH_4_ : alcohol hydroxyl : H_2_O = 1 : 5 : 8. Reaction temperature is 50 °C.

The conversions of NaBH_4_ in different polyol/water system are shown in Fig. 1. It is clearly that the NaBH_4_ conversions in all polyol/water system increase rapidly within 20 min and then stabilize. The NaBH_4_ conversion in methanol/water system is merely 43% after 140 min, which is much lower than that in ethylene glycol/water (88% after 135 min), glycerol/water (92% after 135 min), erythritol/water (96% after 80 min), xylitol/water (90% after 55 min), sorbitol/water (92% after 115 min) and mannitol/water (96% after 105 min) systems. It suggests that polyol can improve the reactivity and H_2_ productivity of alcoholysis of NaBH_4_. In general, the conversion of NaBH_4_ is high in erythritol/water, xylitol/water, sorbitol/water and mannitol/water systems, resulting in a H_2_ productivity of over 90% within 60 min. Considering the relatively high NaBH_4_ conversion and low cost, the erythritol/water system is a promising candidate for hydrogen generation of NaBH_4_ in comparision of xylitol, sorbitol and mannitol. Thus, the further study focused on hydrogen generation from NaBH_4_ in the erythritol/water system.

**Fig. 2:**
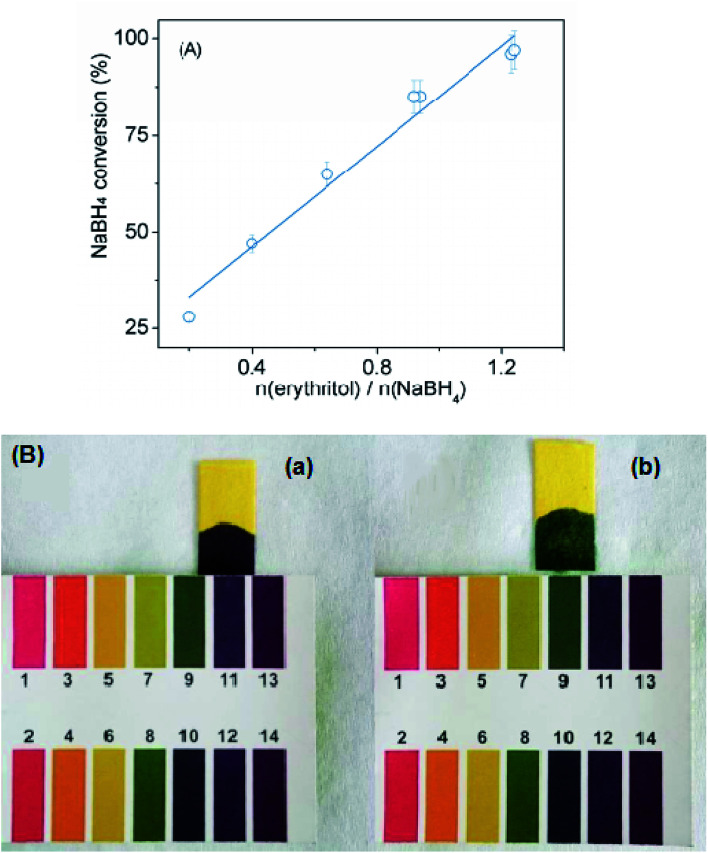
(A) The relationship between NaBH_4_ conversion and mole ratio of erythritol/NaBH_4_. (B) pH values of non gaseous products from (a) methanol/water system and (b) erythritol/water system. Mole ratio of NaBH_4_ : methano/erythritol hydroxyl : H_2_O = 1 : 5 : 8, Reaction temperature is 50 °C.

In order to prove that polyols participate in the reaction, the relationship between conversion and mole ratio of erythritol/NaBH_4_ was systematically studied. As shown in Fig. 2(A), the positive linear relationship between conversion and mole ratio of erythritol/NaBH_4_ indicates that erythritol participates in the reaction rather than acts as catalysts. In order to further compare the difference between methanol/water system and erythritol/water system, the pH values of reaction products in methanol/water system and erythritol/water system were compared in Fig. 2(B). Obviously, the pH value of products in erythritol/water system is about 9, which is significantly lower than that in methanol/water system (pH = 11). It means that the ionization of reaction products in erythritol/water system is inhibited by erythritol, allowing the reaction more complete.

### 3.2 Reaction product characterization

**Fig. 3:**
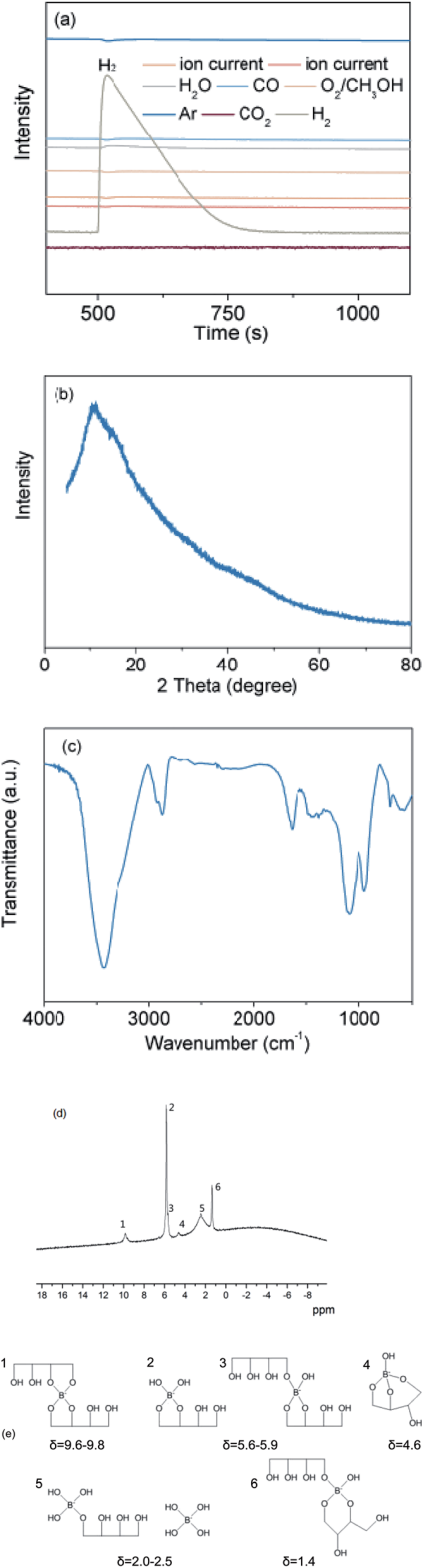
(a) GC chromatograms of gas-phase products. (b) XRD patterns, (c) FTIR spectra and (d) ^11^B-NMR spectra of non gaseous products. (e) The chemical structures corresponding to different chemical shifts. Mole ratio of NaBH_4_ : erythritol : H_2_O = 1 : 1.2 : 8.3. Reaction temperature is 50 °C.

A detailed analysis was carried out to further clarify the chemical structure of the reaction products. Gas-phase products were analyzed using a gas chromatograph (GC). As can be seen from Fig. 3(a), only H_2_ is detected from the gas product. Furthermore, non gaseous products were analyzed by XRD, FTIR and NMR techniques. Prior to all the measurements, the solution after reaction was dried under vacuum and the solid samples were obtained. XRD patterns in Fig. 3(b) show that there is no obvious crystal peak, indicating that the product is amorphous. Thus, it can be inferred that the solid products might be a complex amorphous mixture. The FTIR spectra in Fig. 3(c) show absorption bands corresponding to the vibration absorption of O–H at 3430 cm^−1^ and 1600 cm^−1^. The bands at 2900–3000 cm^−1^, 1100 cm^−1^ and 1300–1500 cm^−1^ can be assigned to stretching vibrations absorption of C–H, C–O and B–O groups, respectively. And the vibration absorption peaks in the range of 1100–1500 cm^−1^ point to typical borate structures, in which boron atoms are coordinated with multiple oxyalkyl groups. Interestingly, the vibration absorption peak of B–O bond is wider than that of standard absorption peak, which might be caused by the different coordination environment of B and O in solid products. In order to further study the coordination environment of B atoms, ^11^B-NMR spectroscopy was employed. As can be seen from Fig. 3(d), 6 peaks could be observed on ^11^B-NMR spectrum, indicating that there are 6 chemical environments of B atoms.

In previous studies, Yoshinobu Miyazaki *et al.* investigated the products from the reaction between erythritol, boric acid (H_3_BO_3_) and sodium tetrahydroxyborate (NaB(OH)_4_).^24^ The specific structures corresponding to different chemical shifts in NMR boron spectra were determined by ^11^B-NMR and DFT calculations, shown in Fig. 3(e). The coordination environment of B atoms represented by the 6 peaks in Fig. 3(d) could be identified using the knowledge from the previous literature report. Apparently, peak-2, 3 are much higher than the others, indicating that structure-2, 3 (*δ* = 5.6–5.9) have a high proportion in the products. In the corresponding structure, a B atom binds to one or two erythritol groups to form a five-membered ring, while the remaining coordination oxygen may come from additional erythritol or hydroxyl groups. It is suggested that water not only acts as solvent but also part of the reaction, and the hydroxyl group of H_2_O goes into the final products.

### 3.3 Reaction mechanism

Over the past decades, the hydrolysis mechanism of NaBH_4_ has been widely studied and a common reaction procedure has been obtained as the following steps:

Step 1: BH_4_^−^ + H^+^ ⇌ H_2_BH_3_

Step 2: BH_4_^−^ + H_2_O ⇌ H_2_BH_3_ + OH^−^

Step 3: H_2_BH_3_ ⇌ H_2_ + BH_3_

Step 4: BH_3_ + 3H_2_O + OH^−^ → B(OH)_4_^−^ + 3H_2_

Based on the analysis of previous experimental results in this work, it is speculated that there might be another subsequent reaction step in the erythritol/water system:

Step 5: B(OH)_4_^−^ + CH_2_OH(CHOH)_2_CH_2_OH → solid products

**Fig. 4:**
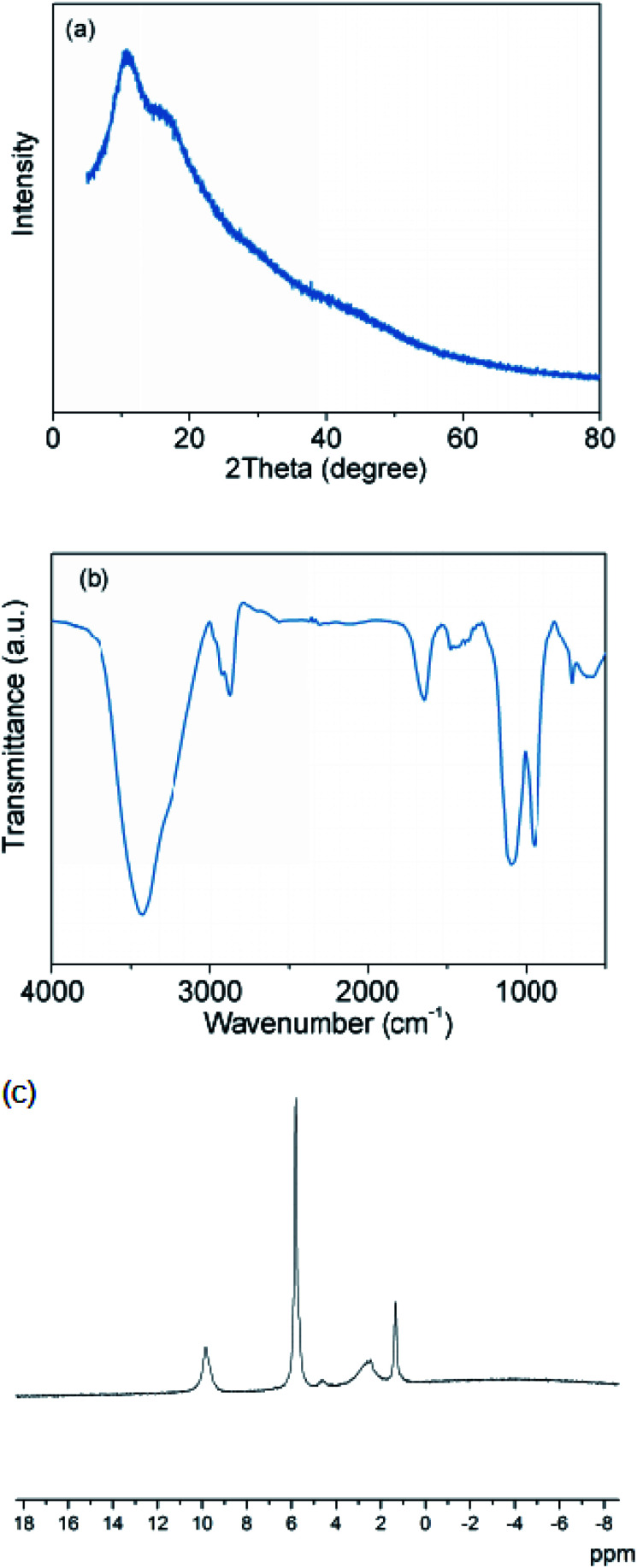
(a) XRD patterns, (b) FTIR spectra and (c) ^11^B-NMR spectra of the reaction products between NaB(OH)_4_ and erythritol/water system. Mole ratio of NaB(OH)_4_ : erythritol : H_2_O = 1 : 1.2 : 8.3. Reaction temperature is 50 °C.

That is, erythritol might further react with NaB(OH)_4_ to produce the final products. To confirm this conjecture, the reaction of NaB(OH)_4_ with erythritol/water system was carried out under the same conditions and the reaction products were comprehensively characterized. As described in Fig. 4, the XRD patterns, FTIR spectra and ^11^B-NMR spectra of the products from the reaction of NaB(OH)_4_ with erythritol/water system are very similar with those between NaBH_4_ and erythritol/water system. It means that erythritol can indeed further react with NaB(OH)_4_ and inhibit the ionization of B(OH)_4_^−^, thus increasing the productivity of reaction.

### 3.4 Optimization of reaction system

In light of above findings, it can be found that the volume of water in the actual reaction system is much larger than the theoretical demand, thus decreasing the gravimetric density of hydrogen generation. Seeking to meet the practical applications of hydrogen energy requirements, the erythritol/water system was further optimized to a quasi-solid phase reaction system *via* adjusting the volume of water. We first optimized the reaction system by turning the mole ratio of the reactants to 1 : 1 : 1. Using this configuration, excellent NaBH_4_ conversion (85%) and gravimetric density of hydrogen storage (3.7 wt%) are observed.

**Fig. 5:**
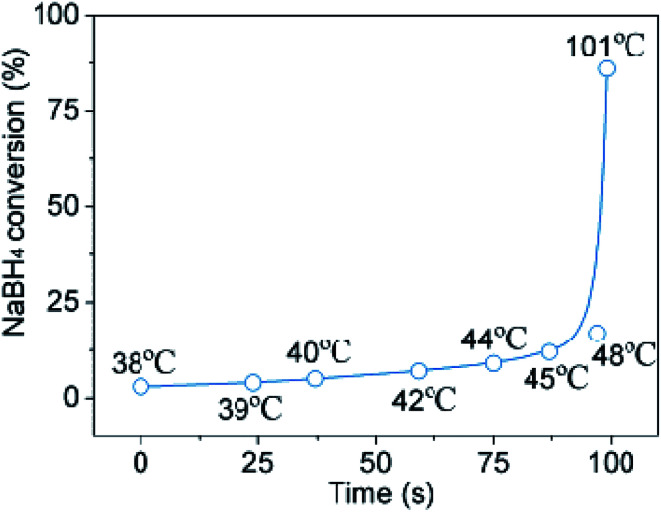
The temperature effect on NaBH_4_ conversion during the reaction. Mole ratio of NaBH_4_ : erythritol : H_2_O = 1 : 1 : 1.

Meanwhile, the temperature of reaction system as the reaction proceeded was tracked (Fig. 5). The temperature curve reveals the presence of a critical temperature point, *i.e.*, 48 °C. Obviously, it takes only about 95 seconds to reach the critical temperature point due to the reduced volume of water. The NaBH_4_ conversion increases rapidly after the critical temperature point. As we adjusted the mole ratio by increasing water volume slowly from 1 : 1 : 0.6 to 1 : 1 : 1.3, monotonic increment in the conversion of NaBH_4_ and the gravimetric density of hydrogen generation were observed. As can be clearly seen from Table 1, when the mole ratio of the reactants is 1 : 1 : 1.3, the NaBH_4_ conversion was as high as 94%, and the gravimetric density of hydrogen storage was 3.9 wt%, close to the requirements for practical applications.

**Table 1:** The optimization of reaction system

Mole ratio of NaBH_4_ : erythritol : H_2_O	NaBH_4_ Conversion (%)	Gravimetric H_2_ storage capacity (wt%)
1 : 1 : 0.6	73	3.3
1 : 1 : 0.8	88	3.8
1 : 1 : 1.1	89	3.8
1 : 1 : 1.3	94	3.9
1 : 1 : 1.7	80	3.2

## 4. Conclusions

In summary, a novel strategy of constructing a polyol/water composite system to induce the alcoholysis of NaBH_4_ while inhibiting the ionization of reaction products was developed. The polyol/water composite systems can be fully reacted (NaBH_4_ conversion of more than 90%) in a relatively short time (within 60 min), especially the erythritol/water system. Erythritol participates in the reaction rather than acts as a catalyst, and the pH value of erythritol/water system is lower than that of methanol/water system. The ionization of reaction products is inhibited by erythritol, thus promoting the conversion of NaBH_4_. Moreover, the chemical structural analysis of reaction products and control group further prove that erythritol can indeed react with NaB(OH)_4_ and inhibit the ionization of B(OH)_4_^−^. On the basis of erythritol/water system, a quasi-solid phase reaction system was developed *via* adjusting the volume of water, which shows an excellent NaBH_4_ conversion (94%) and high gravimetric density of hydrogen storage (3.9 wt%). Briefly, this work provides a new perspective for the design of catalyst free alcoholysis/hydrolysis of NaBH_4_ and further deepens the understanding of mechanism of H_2_ generation from NaBH_4_.

## Conflicts of interest

There are no conflicts to declare.

## Supplementary Material
